# Supplementary material for: HERV6196 as an enhancer with oncogenic potential in rectal cancer
Source: Microbiol Spectr. 2025 Dec 8;14(1):e00788-25. doi: 10.1128/spectrum.00788-25 (PMC12772286; doi:10.1128/spectrum.00788-25)
Supplement: Supplemental material — Fig. S1 and S2; Table S2; Data S1. [file spectrum.00788-25-s0001.pdf]

Supplementary Material

Table of Contents

1. Supplementary Figures 2

- Figure S1. GeneHancer, ChIP-seq and ATAC-seq IGV profiles of HERV6196 as a potential enhancer
- Figure S2.Correlation analysis between HERV6196 and neighboring genes

2. Supplementary Method 4

- Method S1. Cloning of the HERV6196 Enhancer

3. Supplementary Oligonucleotides 6

- Table S1. the differentially expressed HERVs via the DESeq2 package
- Table S2. Primers and Probes Used in This Study

4. Supplementary Sequence Data 7

- Data S1. FASTA Sequence of the HERV6196 Enhancer

1. Supplementary Figures

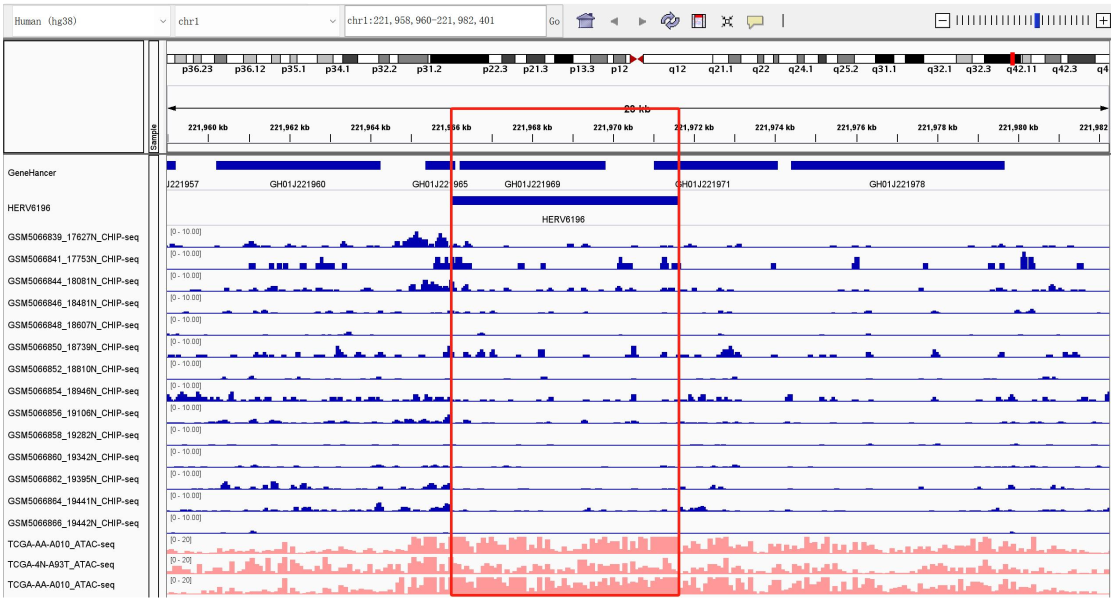

Fig. S1

GeneHancer, ChIP-seq and ATAC-seq IGV profiles of HERV6196 as a potential enhancer. The fourteen ChIP-seq datasets represented adjacent non-cancerous tissues depicted in blue and three ATAC-seq datasets from TCGA depicted in red were used. The red box highlighted the ChIP-seq and ATAC-seq signal peaks in the HERV6196 region.

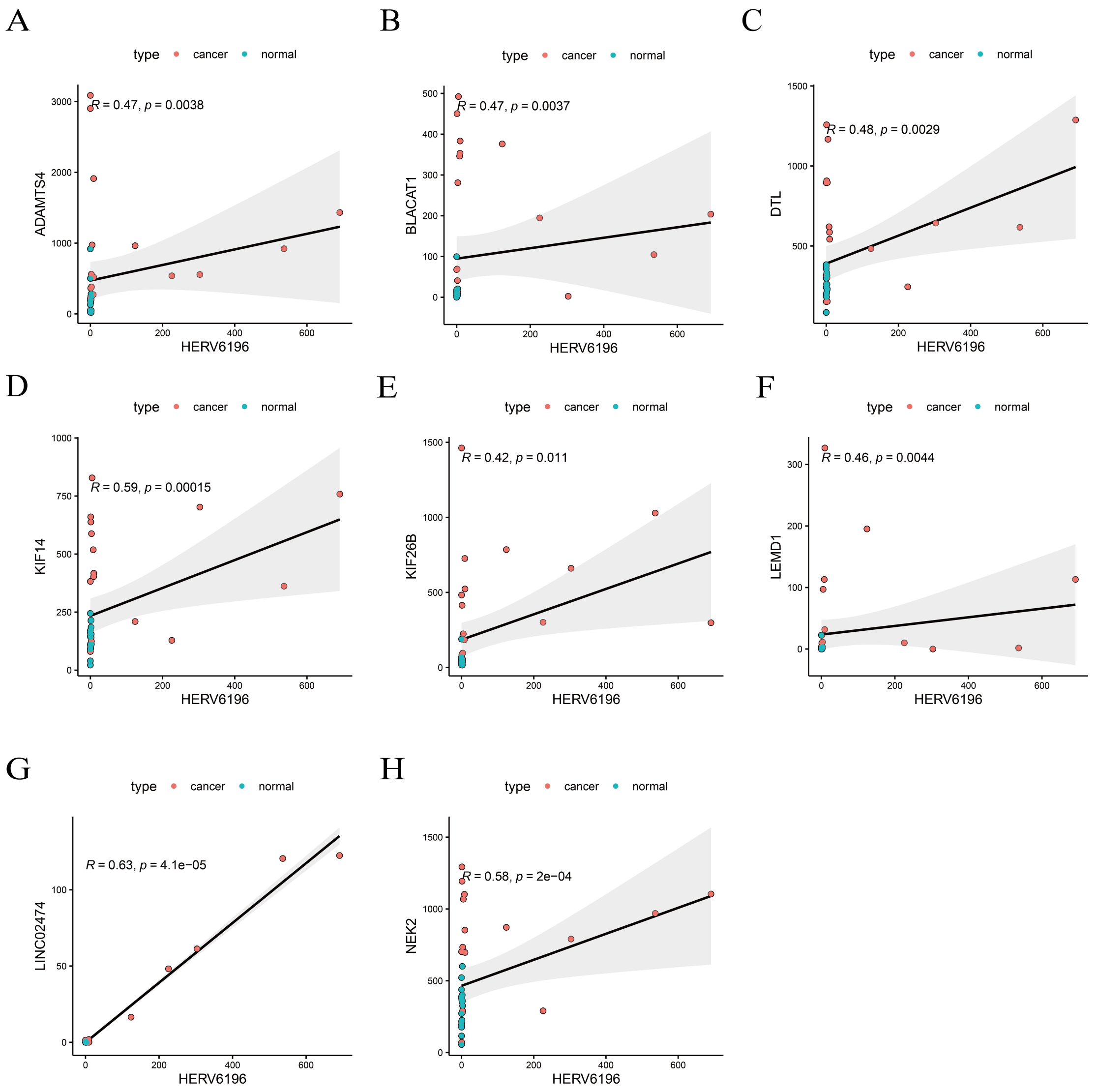

Fig. S2

Correlation analysis between HERV6196 and neighboring genes. (A-I) Correlation analysis between HERV6196 and *ADAMTS4*, *BLACAT1*, *DTL*, *KIF14*, *KIF26B*, *LEMD1*, *LINC02474*, *NEK2*.

2. Supplementary Method

Method S1. Cloning of the HERV6196 Enhancer

1. Enhancer Sequence Information

Name: HERV6196 Enhancer

Genomic Coordinates (GRCh38/hg38): chr1:221,965,963 - 221,971,618

Length: 5,655 bp

Source: Human genomic DNA

Sequence: The full 5,655 bp nucleotide sequence of the enhancer is provided in a separate FASTA file (HERV6196_fasta.fasta).

2. Molecular Cloning Details

Vector: pGL4.23-GV148

Cloning Sites: KpnI (5') and XhoI (3')

Cloning Method: Homologous Recombination using ClonExpress™ II One Step Cloning Kit

3. Primers Used for Cloning

- Construction Primers (for PCR amplification of the enhancer):

Forward Primer (HERV6196-P1):

TTTCTCTATCGATAGGTACCgtgacttgtgcccagatggcctgaagtaactgaagaatcacaaaagaagtgaaagaccctgcccgaccttaactgatgacattccaccattgtgatttgttcctgccccaccttaactgagtg.

(The KpnI site is underlined; lowercase letters represent the homologous arm and enhancer-specific sequence.)

Reverse Primer (HERV6196-P2):

TGCAGATCGCAGATCTCGAGtgtgaagagaccaccaaacaggctttg

(The XhoI site is underlined; lowercase letters represent the homologous arm and enhancer-specific sequence.)

- Colony PCR Identification Primers (for initial screening of positive clones):

Forward Primer (HERV6196-wf9): gttactgaagaatcacaaaa

Reverse Primer (GLprimer2): CTTTATGTTTTTGGCGTCTTCCA

Expected Product Size: 617 bp

4. Validation

The final construct was validated by extensive Sanger sequencing using multiple internal primers to ensure 100% sequence fidelity across the entire enhancer insert.

3. Supplementary Oligonucleotides

- Table S1. the differentially expressed HERVs via the DESeq2 package

Note on Publicly Available Dataset:

The gene expression dataset GSE50760 , GSE104836 and GSE14279 used for comparative analysis in this study has been deposited separately in the submission system.

- Table S2. Primers and Probes Used in This Study

	Forward	Reverse
HERV6196	TTCTCACCCTGATCACGCTT	TTCTAAGAGAGGCGGGCTAGTG
GAPDH	TCGGAGTCAACGGATTTGGT	TTGCCATGGGTGGAATCATA
ADAMTS4	ACATGCTGCACGACAACAGC	GTAGCCGTTGTCCAGGAAGT
DTL	TGGTCTTCACAATACCCTCTTCA	CTTCATTGGCAACTGCTAGTACA
KIF14	CCGACATTACAGATGCACCA	CTTCATTCCTAAGCCTACACC
KIF26B	AGAATAAACCAGCGACCCCA	GCAGACTTCATTCACCCCGTA
LEMD1	ATTGCAGAACCAACTTGAGAAGC	CGCGCAGTAGTCTCTCTCTT
LINC02474	TGCAAGAGGGTCCGAGACAT	ATGTGGTCAAAGGCAGATGCT
NEK2	AAGGGAACCAAGGAAAGGCAA	ACGTTTTGCTTGCCATCCAG

4. Supplementary Sequence Data

Data S1. FASTA Sequence of the HERV6196 Enhancer

```
>1:221965963-221971618
GTGACTTGTGCCCAGATGGCCTGAAGTAACTGAAGAATCACAAAAGAAGTGAAAGAC
CCTGCCCGACCTTAACTGATGACATTCCACCATTGTGATTTGTTCCTGCCCCACCTT
AACTGAGTGATTAACGCTGTGAATTTCCTTCTCCTGGCTCAGAAGCTCCCCCACTGA
GCAGCTTGTGCCCCCACTCCCCCGCCCCTGCCCACCAGAGAACAAACCCCCTTTGAC
TGTAATTTTCCATTACCTTCCCAAATCCTATAAAACGGCCCCACCCCTATCTCCCTT
CATTGACTCTCTTTTCGGACTCAGCCCACCTGCACCCAGGTGAAATAAACAGCCATG
TTGCTCACACAAAGCCTGTTTGGTGGTCTCTTCACACAGATGTGCATGAAATTTGGT
GTGGTGACTCGGATCGGGGGACCTGCCTCAGGAAATCAATCCCCTGTCCTCCTGCTC
TTTGCTCCGTGAGAAAGATCCACCTACGACCTCAGGTCCTCAGACCGACTAGCCCAA
GAAACATCTCACCAATTTCAAATCTGGTAAGCGGCCTCTTTTTACTCTCTTCTCCAA
CTTCCCTCACTATCCCTCAACCTCTTTCTCCTTTCAATCTTGGTGCCACACTTCAAT
CTCTCCCTTCTGTTAATTTCAATTCCTTTCATTTTCTGGTAGAGACAAAGGAGACAC
GTTTTATCCGTGGACCCAAAACTCCGGAGCCGGTCACGGACTGGGAAGGCAGCCTTC
CCTTAGTGTTTAATCATTGCAGGGACACCTCTCTGATTGTTCACCAACGTTTCAAAG
GTGTCAGACCATGCAGGGACGCCTGCCTTAGTCCTTCACCCTTAGCGGCAAGTCCCG
CTTTTCTGGGGGAAGGGCAAGTACCCCAACCCCTTCTCTCCATGTCTCTACCCCTTC
TCTGATTTTCTGGGGCAGGGGCAAGAACCCCTCAACCCCTTCTCCTTCACCCTTAGC
AGCAAGTCCCGCTTTTCTGGGGGAGGGGCAAGTACCCGTCAACCCCTTCTCCTTCAC
CCTTAGCGGTAAGTCCCACTTTTCTAAGGGGCAAGAACGCCCAATCCCTTCTTTCCA
```

CACGCCGACCTCTTATCTCTGTGCCCCAATCCCTTATTTCTGCACCCTGACCTCTTA

TCTCTGTGTCCCAATCCCTTATTTCCGTGCCCCAACCCTTTCTCTGCTTTTCTGGAG

AGCAAGAACCCCCCACCCCTTCTCTGTGTCTCTACTCTTTTCTCTGGGCTTGCCTCC

TTCACTATGGGCAACCTTCCACCTTCCATTCCTCCTTCTTCTCCCTTAGCCTGTATT

CTTAAGAACTTAAAACCTCTTCAACTCTCACCTGACCTAAAATCTAAGCATCTTATT

TTCTTCCGCAATGCCACTTGACCCCAGTACAAACTTGACAGTAGTTACAAATAGCCA

GAAAATGGCGCTTTCAATTTTTCCATCCTACAAGGTCTAAATAATTCTTGTCGTAAA

ATGGGCAAATGGTCTGAGGTGCCTTATGTCCAGGCATTCTTTAACACATCAGTCCCT

TCCTAGTCTCTGTGCCCAGTGCAACTCGTCCCAAATCTTCCTTCTTTCCCTCCCGCC

TGTCCCCTCAGTGCCAACCCCAAGCGTCGCTGAGTCTTTCTAATCTTCCTTTTCTAC

AGACCCATCTGACCTCTCCCCTCCTCGCCAGGCCGAACTAGGTCCTAATTCTTCCTC

AGCCTCCGCTCTTCCACCCTATAATCTTTTTATCACCTCCCCTCCTCACACCTGCTC

CGGCTTACAGTTTCATTCAGTGACTACCCCTCCCCCACCTGCCTAGCAATTTACTCT

TAAAAAGGTGGCTGGAGCCAAAGGCATAGTCAAGGTTAATGCACCTTTTTCTTTATC

CCAAATCAGAAGCATTTAGGCTCTTTTTCATCAAATATAAAAACCGAGCCCAGTTCA

TGGCTCATTCGGCAGCAACCCTGAGATGCTTTACAGCCCTAGACCCTAAAAGGTCAA

AAGGCCGTCTTATTCTCAATATACATTTTATTACCCAATCTGCTCCTGACATTAAAT

AAAACTCCAAAAATTGGAATCTGGCCCTCAAACCCCACAACAGGACTTAATTAACCT

CACTTTCAAGGTGTACAATAACAGAAAAAAGTTGCAATTCCTTGCCTCCACTGTGAG

ACAAACCCCAGCCACATCTCCAGCACACAAGAACTTCCAAACACCTAAACCGCAGTG

GCCAGGGGTTCCTCCAAAACCTCCTCCCCCAGGAGTTTGCTACAAGTGCCAGAAATC

TGACCACCAGGCCAAGGAATGCCTGGAGCCCAGGATTCTTCCTAAGCCGTGTCCCAT

CTGTGCGGGACCCCACTGAAAATCGGACTGTTCATCTCACCTGGCAGCCACTCCCAG

AGCCCGTGGAACTCTGGCCCAAGGCTCTCTGACTGACTCCTTCTTGGCTTAGCGGCT

GAAGACTGACACTGCCCGATCGCCTCAGAAACCCCGTAGACCATCACGGACGCCGAG

CTTTAGTTAACTCTCACAGTGGAGGGTAAGTCCGTCCCCTTCTTAATCAATACGGAG

GCTACCCACTCCACATCACCTTCTTTTCAAGGGCCTGTTTCCCTTGCTTCCATAACT

GTTGTGGGTATTGACAGCCAGGCTTCTAAACCTCTTAAAACTCCCCAACTCTGGTGC

CAACTTAGACAATACTCTTTTAAGCACTCCTTTTTAGTTATCCCCACCTGCCCAGTT

CCCTTATTAGGCTGAGACACTTTAAATTATCTGCTTCCCTGACTATTCCTGGACTAC

AGCTACATCTCATTGCCACCCTTCTTCCCAATCCAAATCCTCCTTTGCGTCCTCCTC

TTGTATTCCCCCACCTTAACCCACAAGTATAAGATACCTCTACTCCCTCCTTGGTGA

CCGATCATGCACCCCTTACCATCTCATTAAAACCTAATCATTCTTACCCCCTCAATG

TCAAGATCCCATCCCGCAGCACGCTTTAAAAGGATTAAAGCCTGTTATCACTCGCCT

GCTATAGCATGACCTTTTAAAGCCTATAAACTCTCCTTACCATTCCCCCATTTTACC

TGTCCTAAAACCAGATAAACCTTACAAGTTAGTTCAGGATCTGCACCTTATCAACCA

AATTGTTTTGCCTATCCACCCCGTGGTGCCAAACCCATACACTCTCCTATCCTCAAT

ACCTCCCTCTACTACCCATTATTCTGTTCTGGATCTCAAACATGCTTACTTTACTAT

TCCTTTGCACCCTTCATCCCAGCCTCTCTTTGCTTTCACTTAGACTGACCCTGACAC

CCATTAGGCTCAGCAATTACCTGGGCTGTACTGCCGCAAGGCTTCACAGACAGACCC

CATTTCAGTCAAGCCCAAATTTCATCCTCATCTGTTACCTATCTCGGCATAATTCTC

GTAAAAACACACTTGCTCTCCCTGCTGATCTTGTCCGATTAATCTCCCAAACCTCAA

TCCCTTACAAAACAACAACTCCTTTCCTTCCTAGGCATGGTTAGTGCGGTCAGAATT

CTTACACAAGAGCCAGGACCACACCCTGTAGCCTTTCTGTCCAAACAACTTGACCTT

ACTGTTTTAGCCTAGCCCTCATGTCTGTGTGCAGCAGCTGCCACTGCTTTCATAATT

TTAGAGGCCCTCAAAATCACAGACTATGCTCAACTCACTCTCTACAGTTCTCATAAC

TTCCAAAATCTATTTTCTTCCTCATACCTGACTCATATACTTTCTGCTCCCTGGCTC

CTTCAGCTGTACTCACATTTTGTTAAGTCCCACAATTACCATTGTTCCTGGCCCAGA

CTTCAATCCGGCCTCCCACATTATTCTAGATACCACACCTGACCCCCATGACTGTAT

CTCTCTGATCCACCTGATGTTCATCCCATTTCCCCACATTTCCTTCTTCCCTGTTTC

TCACCCTGATCACGCTTGATTTATTGATGGCGGTTCCACCAGGCCTAATCGCCACAC

ACCAGCAAAGGCAGGCTATGCTATAGTAGAAGCCACTAGCCCGCCTCTTAGAACCTT

TCATTTCCTTTCCATTGTAGAAATCTATCCTCAAGGAAATAACTTCTCAGTGTTCCA

TCTGCTATTCTACTACTCCTCAATGATTATTCAGGCCCACTACCTTCTCTACACATC

AATCTGGAGGATTTGCCCCCACCCAGGACTGGCAAATTAGCTTTACTCAACATGCCC

CGAGTCACAAAAACTAAAATACCTCTTATTCTAAGTAGACACTTTCACTAGTTAGGT

AGAGGCCTTTCCTACAGGGTCTGAGAAGGCCACCGCAATCCTTTCTTCCCTTCTGTC

AGACATAATTCCTCAGTTTAGCCTTCCCACCTCTACACAGTCTGATAACAGACCAGT

CTTTATTAATCAAATCAGCCAAGCATTTTCTCAGGCTCTTAGTATTCAGTGACAGAC

TAATGGTCTATTAAAAACACACCTCACCAAGCTCAGCCACCAACTTAAAAAGGACTG

GACAATACTTTTACCACTTTCCCTTCTCAGAATTCAGCCTGTCCTTGGAATGCTGCA

GGCTACAGCCCATTTAAGCTCCTGTATAGATGCTCCTTTTTATTAGGCCCCAGTCTC

ATTCCAGACACCAGACCAACTTAGACTGTGCCCCCAAATAACTTGTCATCCGTACTA

TCTTCTATCTAGTCATACTCCTATTCACTGTTCTCAACTACTCATACATGCCCTGCT

CTTGTTTACACTGCCGGTTTACACTGTTTCTCCAAGCCATCACAGCTGATATCTCCT

CCTGCTATCCCCAAACTGCCACTCTTAACTCTTGAAGTAAATAAATAATCTTTGCTG

GCAGGACTATGCTGAACCTCCTTAGGCACTCTCTAATTAGATGTCCTAGGTCCTCCC

AATTCTTAGTCTTTTTATACCTGTTTTTCTCCTTCTCTTATTCCATTTAGTTTTTCA

ATTCATACAAAACCGTATCCAGGCCATCACCCATCACTCTATATGACAAATGTTTCT

TCTAACAACCCACAATATCAACCCTTACCACAAGACCTCCCTTCAGCTTAATCTCTC

CCACTCTAGGTTCCCACGCTGCCCCTAATCCCGCTTGAAGCAGCCCTGAGAAACATC

GCCCATTCTCTCTCCATACCACCCCCAAAAAATTTTCGCCGCCCCAACACTTCAACA

CTATTTTGTTTTATTTTTCTTATTAATATAAGAAGGCAGGAATGTCAGGCCTCTGAA

CCCAAGCCAAGCCATCACATCCCCTGTGACTTGCACGTATGCACGTATGCACCTAGA

TGGCCTGAAGTTACTGAAGAATCACAAAAGAAGTGAAAAGGCCCTGCCCCGCCTTAA

CTGATGACATTCCACCATTGTGATTTGTTCCTGCCCCACCTTAACTGAGTGATTAAC

CCTGTGAATTTCCTTCTCCTGGCTCAGAAGCTCCCCCACTGATCAGCTTGTGACCCC

CGCCCCTGCCCACCAGAGAACAAACCCCCTTTGACTGTAATTTTCCATTACCTTCCC

AAATCCTATAAAACGGCCCCACCCTTATCTCCCTTCGCTGACTCTTTTCGGACTCAG

CCCATCTGCACCCAGGTGAAATAAACAGCCATGTTGCTCACACAAAGCCTGTTTGGT

GGTCTCTTCACA
